# Deep Learning-Driven Identification and Experimental Validation of COX-2 and ERK1/2 as Anti-Inflammatory Targets of Avenanthramides

**DOI:** 10.3390/nu18203339

**Published:** 2026-10-12

**Authors:** Jingjie Zhang, Tengyu Li, Chi Yan, Zeran Wang, Zhiyao Zhao, Yonghui Yu, Ding Wang

**Affiliations:** 1 Key Laboratory of Geriatric Nutrition and Health, Ministry of Education, Beijing Technology and Business University, Beijing 100048, China; jingjiezhang@btbu.edu.cn (J.Z.); 15028307047@163.com (T.L.); 17721500254@163.com (C.Y.); 18986364853@163.com (Z.W.); 2 China-Canada Joint Lab of Food Nutrition and Health, Beijing Technology and Business University, Beijing 100048, China; 3 Key Laboratory of Special Food Supervision Technology for State Market Regulation, Beijing Technology and Business University, Beijing 100048, China; 4 School of Computer and Artificial Intelligence, Beijing Technology and Business University, Beijing 100048, China; foz.ding@gmail.com

**Keywords:** deep learning, anti-inflammatory virtual screening, molecular docking, avenanthramides, COX-2, ERK1/2

## Abstract

Background: Chronic low-grade inflammation underlies numerous chronic diseases, yet the molecular targets of dietary bioactive compounds remain largely unknown. Methods: We developed a deep learning model integrating heterogeneous network propagation, stacked denoising autoencoders, and a CNN classifier to predict inflammation-related targets of food-derived compounds. The model was validated by cross-validation and on eight compounds from distinct structural categories. Avn A, Avn B, and Avn C were then input into the model to predict their anti-inflammatory targets, followed by network analysis, docking, and cellular assays for validation. Results: The model achieved a mean AUC of 0.9587 and an AUPR of 0.9590, with validation on eight structurally diverse compounds confirming its generalizability. For Avn A, Avn B, and Avn C, 118 overlapping anti-inflammatory targets were predicted. Network analysis and KEGG pathway enrichment identified ERK1/2 as a key node within the MAPK and PI3K-AKT signaling pathways, while COX-2 was prioritized for further validation based on its high prediction score. Molecular docking revealed that Avns bound at the cyclooxygenase channel entrance of COX-2 with binding energies of −7.7 to −8.0 kcal/mol, and at the ATP-binding pockets of ERK1/2 with binding energies of −7.0 to −7.7 kcal/mol. In LPS-stimulated macrophages, Avns dose-dependently inhibited COX-2 enzymatic activity and PGE2 production, attenuated ERK1/2 and c-Fos phosphorylation, and suppressed IL-6 and IL-1β expression. Conclusions: Our deep learning model successfully identified COX-2 and ERK1/2 as key mediators of the anti-inflammatory effects of Avns, revealing a dual mechanism involving COX-2 inhibition and ERK/c-Fos pathway blockade. This study provides new evidence for the molecular targets of Avns and demonstrates the utility of deep learning-based approaches for target discovery of dietary bioactive compounds.

## 1. Introduction

Chronic low-grade inflammation, characterized by a persistent but modest elevation of circulating pro-inflammatory cytokines such as tumor necrosis factor-α (TNF-α) and interleukin-6 (IL-6) in the absence of detectable infection, is strongly associated with the development of diverse chronic diseases, including cardiovascular disorders, type 2 diabetes, obesity, and certain cancers [1]. A recent population-based cohort study of over 270,000 adults reported that higher aggregated inflammation scores correlated with a 36% greater risk of cardiometabolic multimorbidity and an earlier onset by nearly 13 months [2], highlighting the contribution of persistent inflammation to chronic disease pathogenesis. Conventional anti-inflammatory pharmacotherapy, particularly non-steroidal anti-inflammatory drugs, carries well-documented gastrointestinal and cardiovascular risks that limit their long-term use [3]. This safety concern has prompted growing interest in identifying effective and safe anti-inflammatory agents from dietary sources. Food-derived bioactive compounds have shown considerable potential in modulating inflammatory responses. Various food-derived compounds, such as ferulic acid [4,5] and curcumin [6,7], have been widely investigated, with reported effects including suppression of pro-inflammatory mediators and attenuation of NF-κB and MAPK signaling. However, for the majority of food-derived bioactives, the anti-inflammatory targets remain largely unexplored, which limits both the mechanistic understanding of their health benefits and the rational development of targeted nutritional interventions.

Traditional experimental methods for identifying compound-target interactions (CTIs), including affinity chromatography, protein microarrays, and high-throughput screening, are often labor-intensive, expensive, and time-consuming, and are not well-suited for systematically screening the targets of small-molecule compounds [8]. In recent years, deep learning-based approaches have emerged as powerful tools for CTI prediction by leveraging large-scale heterogeneous biological data and learning complex nonlinear relationships from chemical and protein features. A variety of models, such as DeepDTA [9] and DeepConv-DTI [10], both of which are based on convolutional neural networks, have achieved impressive predictive performance in drug-target interaction prediction. However, these models are generally trained and validated on drug-like molecules and pharmacological targets, and their applicability to food functional factors and inflammation-related targets has not been fully explored. Recent studies have begun to apply machine learning approaches to food-derived compound screening. For example, graph convolutional networks have been employed to predict anticancer molecules within foods based on the similarity between bioactive food molecules and FDA-approved drugs [11]. Fingerprint and graph fusion learning have been integrated to identify TLR4 inhibitors from medicinal food plants [12]. Random forest models have also been utilized to screen DPP4-targeting nutritional metabolic regulators from food-derived chemical space [13]. In the context of anti-inflammatory peptides, several computational predictors have been developed, including DeepAIP based on pre-trained protein language models and contextual self-attention networks [14], the NeXtMD stacked framework integrating machine learning and deep learning components [15], and ensemble learning models with multi-feature fusion strategies [16]. Despite these preliminary efforts on anti-inflammatory peptides and other bioactivities, a comprehensive and systematic prediction of food-derived small-molecule phytochemicals specifically targeting inflammation-related pathways, covering both known and potential anti-inflammatory targets, remains largely unexplored. There is a clear need for customized predictive models that can bridge the gap between food bioactives and their molecular targets in the context of inflammatory regulation.

Avenanthramides (Avns) are a group of phenolic alkaloids uniquely found in oats [17]. Among the over 40 identified Avns, Avn A, Avn B, and Avn C are the most abundant and have been extensively studied for their anti-inflammatory, antioxidant, and antiproliferative activities [18]. Mechanistic studies have demonstrated that Avns can modulate multiple inflammation-related signaling pathways, leading to reduced expression of pro-inflammatory mediators such as COX-2, IL-6, and IL-1β [19,20]. However, whether Avns exert these effects through physical binding to specific protein targets or via upstream regulatory mechanisms has not been experimentally resolved. To date, only a handful of targets of Avns have been identified, mainly in the field of cancer, such as DDX3 [21] and KDM4C [22]. The anti-inflammatory targets of Avns remain largely unknown, representing a critical bottleneck for understanding their molecular basis of action and translating these findings into practical applications. Therefore, we developed a deep learning-based model integrating random walk with restart (RWR) on a heterogeneous network, stacked denoising autoencoders (DAE), and a one-dimensional convolutional neural network (1D-CNN) to predict inflammation-related targets of food-derived phytochemicals. The model was applied to Avns, and the top-ranked predictions were validated through bioinformatics analysis, molecular docking, and cellular assays. This integrated computational–experimental strategy offers a promising approach for uncovering the molecular targets of dietary bioactive compounds and elucidating their anti-inflammatory mechanisms.

## 2. Materials and Methods

### 2.1. Construction of the Inflammation-Related Target Prediction Model

#### 2.1.1. Data Collection and Dataset Preparation

Inflammation-related targets were retrieved from the DrugBank database [23] using keywords including “inflammation,” “inflammatory response,” and “immune response”. After manual curation to remove incomplete or duplicate entries, 742 targets were retained. These targets were then used to reversely retrieve known interacting compounds from DrugBank, yielding 540 compounds and 2398 compound-target interaction pairs, which served as the positive sample set. All compounds in the positive set have at least one known interaction with an inflammation-related target. The negative sample set was constructed by random sampling from the full combinatorial space of compounds and targets, excluding the 2398 known CTIs from DrugBank. These randomly sampled pairs were treated as putative non-interacting pairs under the assumption that most unrecorded pairs are non-interacting; experimentally validated negatives are rarely available, and random sampling is a widely used approximation.

#### 2.1.2. Heterogeneous Network Construction and RWR-Based Feature Extraction

Three matrices were constructed for compounds and three for targets. For compounds, these are (i) compound–compound structural similarity derived from chemical structure information, (ii) compound–disease associations, and (iii) compound–compound similarity based on shared targets (i.e., compounds sharing the same target proteins). For targets, these are (i) target–target structural similarity derived from target sequence/structure information, (ii) target–disease associations, and (iii) target–target similarity based on shared compounds (i.e., targets sharing the same interacting compounds). To quantify the strength of association between biological entities, the Jaccard similarity coefficient was used to measure pairwise similarity between compounds and between proteins [24]. For two binary vectors $X_{i}$ and $X_{j}$ representing two molecules’ interaction profiles, the Jaccard similarity is defined as:

$$J\left(X_{i},X_{j}\right)=\frac{\left|X_{i}\cap X_{j}\right|}{\left|X_{i}\cup X_{j}\right|}=\frac{M_{11}}{M_{01}+M_{10}+M_{11}}$$

where $M_{11}$ is the number of dimensions where both are 1, and $M_{01}$ and $M_{10}$ are the counts of dimensions where the two vectors differ (0/1 or 1/0, respectively). The compound-compound and target-target similarity matrices were computed using the Jaccard coefficient from the binary compound-protein association matrix. For a given association matrix ***M***, each row vector represents the interaction profile of a compound, and each column vector represents the interaction profile of a protein. Pairwise Jaccard similarity was computed between these vectors using *S* = 1 − *D_Jac_*, where *D_Jac_* was calculated with the pdist function in SciPy (version 1.10.0, SciPy Developers, Austin, TX, USA). The compound-target similarity and shared-compound target similarity matrices were also derived from the same association matrix using Jaccard similarity between the corresponding interaction profiles. The target-disease and protein-disease association matrices were constructed from disease annotation data.

To capture global topological information and mitigate data sparsity, the RWR algorithm was applied to the constructed heterogeneous network [25,26]. Given a row-normalized transition probability matrix ***P***, the RWR iterative update is defined as:

$$Q_{t+1}=\left(1-c\right)PQ_{t}+cR$$

where c is the restart probability and ***R*** is the restart matrix, set to the identity matrix ***I*** in this study to obtain feature representations for all nodes. The iteration was repeated until the Frobenius norm of the difference between successive ***Q*** matrices fell below a predefined threshold [27,28]. For each input similarity network (e.g., compound structural similarity and protein sequence similarity), RWR was independently applied to generate diffused feature matrices, which were subsequently concatenated and log-transformed to compress the numerical range and enhance feature expressiveness:

$$Q_{joint}=\left[Q^{\left(1\right)},Q^{\left(2\right)},\ldots ,Q^{\left(k\right)}\right]$$

$$Q_{final}=\log\left(Q_{joint}+\frac{1}{N}\right)-\log\left(\frac{1}{N}\right)$$

#### 2.1.3. Stacked Denoising Autoencoder for Feature Optimization

Although the RWR algorithm generated feature matrices containing global topological information, the resulting feature dimensions remained high and potentially contained redundant information and noise. To obtain more compact and robust low-dimensional feature representations, an unsupervised deep learning-based feature learning model was introduced.

A DAE extends the standard autoencoder by corrupting the input with noise and training the network to reconstruct the original clean input from the corrupted version [29]. This mechanism forces the model to capture essential statistical structures rather than simply memorizing the input, thereby overcoming the limitation of standard autoencoders that tend to learn identity mappings. The DAE consists of two components: an encoder that maps the high-dimensional input *x* to a low-dimensional latent representation *h*:

$$h=f\left(W_{x}+b\right)$$

and a decoder that reconstructs the latent representation back to the original space:

$$y=g\left(W^{\prime }h+b^{\prime }\right)$$

where *f* and g are nonlinear activation functions, ***W*** and ***W′*** are weight matrices, and ***b*** and ***b′*** are bias vectors. The training objective minimizes the reconstruction error between the input *x* and the output *y*, using the mean squared error loss:

$$L\left(x,y\right)=\frac{1}{2}{\left\|x-y\right\|}^{2}$$

To enhance robustness, additive Gaussian noise was injected into the input:

$$\tilde{x}=x+\lambda \cdot \varepsilon ,\varepsilon \sim N\left(0,I\right)$$

where *λ* controls the noise intensity. The model was trained to reconstruct the original clean input x from the corrupted version $\tilde{x}$, forcing the network to learn features robust to minor perturbations.

To extract deeper abstract features, a stacking strategy was adopted. A greedy layer-wise training strategy was employed [29]: the first DAE was trained, its parameters were frozen, and its hidden layer output served as input to the next DAE. By cascading multiple DAEs, the feature dimensionality was progressively reduced. Specifically, the compound feature extraction network ultimately reduced the feature dimension to 100, and the protein network to 400. The Softplus function, $f\left(x\right)=ln(1+e^{x})$, was used as the activation function, and the RMSprop optimizer was applied to accelerate convergence.

Through this stacked denoising autoencoder, the high-dimensional sparse features generated by RWR were transformed into low-dimensional dense latent feature vectors, preserving critical topological information while eliminating noise, and providing high-quality input features for the subsequent supervised classification task.

#### 2.1.4. 1D-CNN Classifier and Performance Evaluation

The learned drug and protein feature vectors were concatenated into a 1D feature vector of length 500, which served as input to a 1D convolutional neural network (CNN) classifier [30]. The architecture consisted of: (i) a 1D convolutional layer with 4 filters, kernel size 4, and stride 1 (ReLU activation); (ii) a max-pooling layer with pool size 2 and stride 2; (iii) a dropout layer with rate 0.5; (iv) a fully connected dense layer with 128 neurons (ReLU activation); and (v) an output layer with 1 neuron and sigmoid activation, yielding the interaction probability. The model was trained with the binary cross-entropy loss function:

$$L=-\frac{1}{N}\sum_{i=1}^{N}\left[y_{i}\log\left(\hat{y_{i}}\right)+\left(1-y_{i}\right)\log\left(1-\hat{y_{i}}\right)\right]$$

where $y_{i}$ is the true label of the i-th sample and $\hat{y_{i}}$ is the predicted probability. The Adam optimizer was employed with an initial learning rate of 0.002.

**Figure 1:**
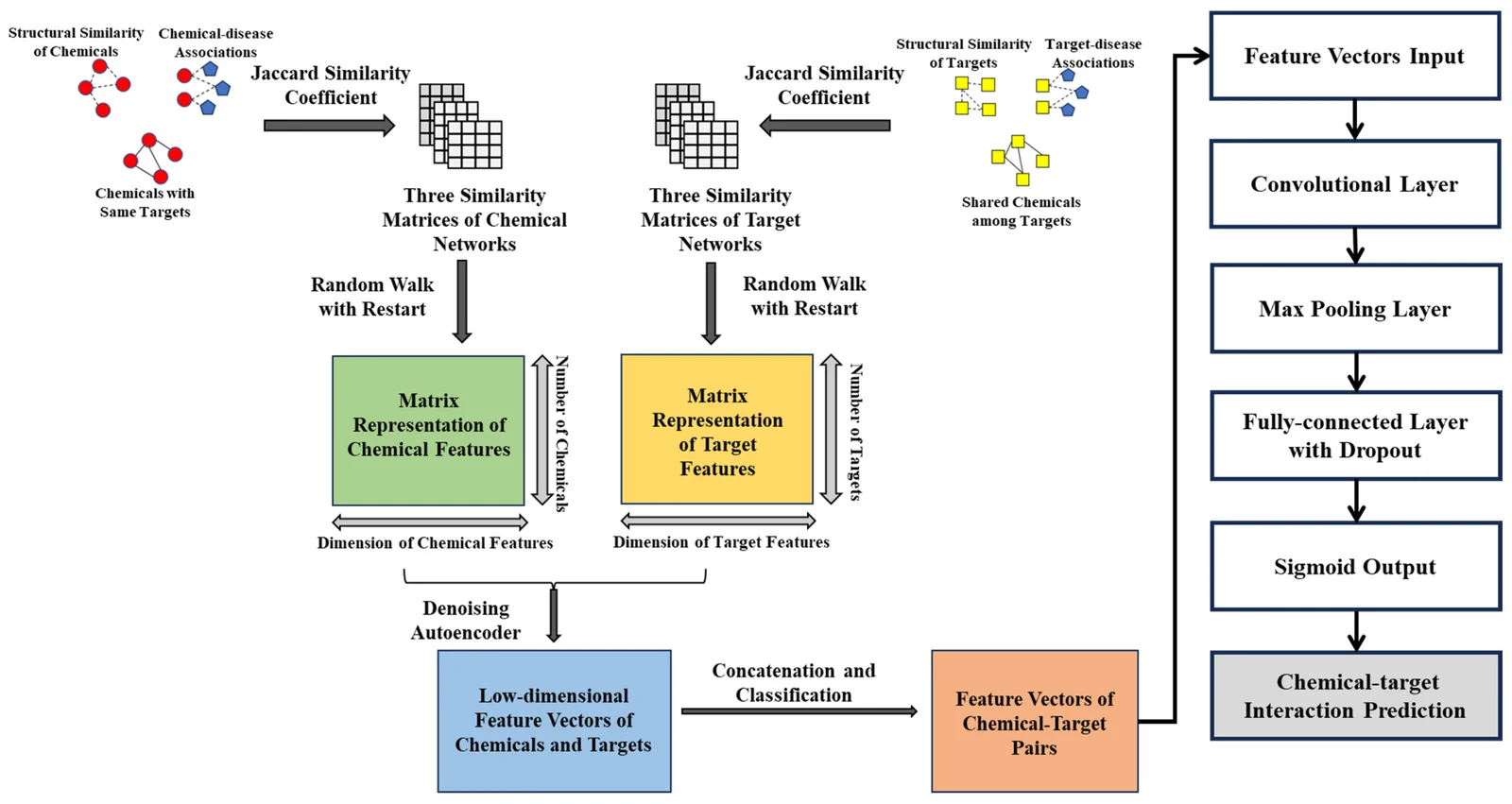
The overall framework of the CTI prediction model developed in this study. As shown, the workflow begins with the construction of three types of similarity matrices for compounds and targets, based on structural similarity, disease associations, and shared chemicals, with the random walk with restart algorithm applied in the process. These matrices were subsequently processed by a stacked denoising autoencoder for nonlinear dimensionality reduction and feature optimization. The optimized features were then used as inputs to a one-dimensional convolutional neural network, which was trained to predict compound-target interactions.

Model performance was evaluated by stratified 10-fold cross-validation. The dataset was randomly partitioned into 10 mutually exclusive subsets, with each subset used once as the test set and the remaining nine as the training set [31]. The area under the receiver operating characteristic curve (AUC) and the area under the precision-recall curve (AUPR) were adopted as evaluation metrics [32] (Figure 1). The code and positive CTI pairs are available at https://github.com/zjjamelia/CTI-prediction-model (accessed on 8 August 2026).

#### 2.1.5. Preliminary Model Validation with Known Food Functional Factors

To preliminarily validate the reliability of the trained model, eight common food functional factors, quercetin (flavonoid), epigallocatechin gallate (EGCG, catechin), artemisinin (sesquiterpene lactone), ferulic acid (phenolic acid), eicosapentaenoic acid (EPA, omega-3 polyunsaturated fatty acid), β-carotene (terpenoid), caffeine (alkaloid), and limonene (monoterpene), were selected [33]. These compounds cover a broad spectrum of chemical categories and dietary sources, ensuring a comprehensive test of the model’s predictive capability. Their canonical SMILES strings were input into the trained model to generate predicted interaction probabilities against all target proteins in the dataset. For each compound, the top 5 predicted targets (ranked by interaction probability) were selected and subjected to molecular docking to assess binding affinities (see Section 2.3 for docking procedures). On one hand, strong binding affinities would corroborate the model’s predictive reliability. On the other hand, the resulting compound-target pairs with high docking scores also revealed previously unrecognized potential targets for these food functional factors, offering new directions for future experimental validation.

### 2.2. Prediction and Analysis of Potential Anti-Inflammatory Targets of Avns

#### 2.2.1. Target Prediction and Union Analysis

The canonical SMILES strings of Avn A, Avn B, and Avn C were obtained from the PubChem database [34] (https://pubchem.ncbi.nlm.nih.gov/, accessed on 8 August 2026) and input into the prediction model. The top 100 predicted targets per monomer were retained. A Venn diagram was generated using Venny 2.1 (https://bioinfogp.cnb.csic.es/tools/venny/, accessed on 8 August 2026) to obtain the union set of all potential targets across the three monomers.

#### 2.2.2. PPI Network Construction and Core Target Identification

The union targets were imported into the STRING database [35] with “Homo sapiens” selected. A protein–protein interaction (PPI) network was constructed with a high confidence score (0.9), and disconnected nodes were hidden. The network was analyzed using Cytoscape 3.8.0 [36]. Core targets were identified as those with degree, betweenness centrality, and closeness centrality all exceeding the respective median values.

#### 2.2.3. GO and KEGG Enrichment Analysis

Gene Ontology (GO) enrichment analysis, covering biological process (BP), cellular component (CC), and molecular function (MF), and Kyoto Encyclopedia of Genes and Genomes (KEGG) pathway enrichment analysis were performed on the predicted anti-inflammatory targets of Avns using Metascape [37] (version v3.5.20260701, https://metascape.org/, accessed on 8 August 2026) with the default background gene set. Multiple-testing correction was applied using the Benjamini–Hochberg procedure, and a Q value (adjusted *p* value) < 0.05 was set as the significance threshold. Results were visualized as bar charts and bubble plots, respectively.

### 2.3. Molecular Docking

Molecular docking was performed to assess the binding capability between candidate compounds and their predicted target proteins using AutoDock 4.2.6 (The Scripps Research Institute, La Jolla, CA, USA) [38]. The 3D structures of all ligand compounds, including the eight food functional factors (quercetin, EGCG, artemisinin, ferulic acid, EPA, β-carotene, caffeine, and limonene) and Avn A/B/C, were downloaded from PubChem in SDF format and converted to PDBQT format with Gasteiger charges assigned using OpenBabel (version 3.1.0). The crystal structures of the receptor proteins were retrieved from the RCSB Protein Data Bank (PDB; https://www.rcsb.org/, accessed on 8 August 2026). Water molecules were removed and polar hydrogens were added using AutoDock Tools, and the prepared structures were saved in PDBQT format. Specifically, for the eight food functional factors, the top 5 predicted targets per compound (40 compound-target pairs in total) were subjected to docking; for Avn A, Avn B, and Avn C, docking was performed against the core target proteins COX-2 (PTGS2), MAPK3 (ERK1), and MAPK1 (ERK2).

Docking against MAPK3 and MAPK1 was carried out using AutoDock 4.2.6. For MAPK3 (human ERK1, PDB: 2ZOQ), the grid box was centered at X = 31.094, Y = 7.273, Z = 18.538, with 40 grid points in each of the X, Y, and Z dimensions and a grid spacing of 0.375 Å (total 64,000 grid points). The binding site was defined by the co-crystallized ligand 5ID. For MAPK1 (human ERK2, PDB: 4QTA), the grid box was centered at X = 30.671, Y = 56.509, Z = 32.370, with 26, 64, and 32 grid points in the X, Y, and Z dimensions, respectively, and a grid spacing of 0.375 Å (total 57,915 grid points). The binding site was defined by the co-crystallized ligand 38Z, and the grid box was narrowed to this region to perform site-directed docking. For each ligand–receptor pair, 30 independent docking runs were performed, and the conformation with the lowest binding free energy (kcal/mol) was selected for subsequent analysis.

Docking against COX-2 was performed using the CB-Dock3 web server, and the receptor structure was prepared with the haem group (COH) explicitly retained. Among the cavities detected by CB-Dock3, the one corresponding to the cyclooxygenase channel entrance was selected for docking analysis, rather than the cavities on the protein surface. For each ligand–receptor pair, the conformation with the lowest binding free energy within this cavity was selected for subsequent analysis.

Protein–ligand interactions were profiled using the PLIP online platform (https://plip-tool.biotec.tu-dresden.de/, accessed on 8 August 2026) [39], and binding poses were visualized with PyMOL 2.5.0 (Schrödinger, LLC, New York, NY, USA).

### 2.4. In Vitro Experimental Validation

#### 2.4.1. Cell Culture

RAW264.7 murine macrophages were obtained from Procell Life Science and Technology Co., Ltd. (Wuhan, China). Cells were cultured in high-glucose DMEM supplemented with 10% fetal bovine serum and 1% penicillin-streptomycin at 37 °C in a 5% CO_2_ incubator. Cells were passaged upon reaching 80~90% confluence by gentle pipetting (without trypsin), at a split ratio of 1:4, and all experiments were performed with cells in the logarithmic growth phase.

#### 2.4.2. CCK-8 Cell Viability Assay

The cytotoxicity of Avns on RAW264.7 macrophages was assessed using the CCK-8 assay. Cells were seeded into 96-well plates at a density of 5 × 10^4^ cells/well (100 µL per well) and cultured for 24 h. Subsequently, the medium was replaced with fresh complete medium containing graded concentrations of Avns (5, 10, 20, 40, 80, and 160 μg/mL), and cells were incubated for another 24 h. A blank control (medium only, without cells) and a control group (cells without Avns treatment) were included, and each concentration was tested in 3 replicate wells. Following treatment, the supernatant was discarded, and 100 µL of serum-free DMEM containing 10% CCK-8 reagent was added to each well. The plates were incubated at 37 °C for 1.5 h in the dark. The absorbance was recorded at 450 nm using a microplate reader (BioTek Instruments, Inc., Winooski, VT, USA), with the blank control used for background subtraction. Cell viability was calculated as a percentage of the untreated control group.

#### 2.4.3. Experimental Design

To evaluate the anti-inflammatory effect of Avns, RAW264.7 cells in the logarithmic growth phase were seeded into culture plates (6-well or 12-well plates depending on the assay) and allowed to adhere overnight. Cells were then divided into the following five groups: blank control (untreated); LPS model (2 μg/mL LPS); Avns low-dose (2 μg/mL LPS + 10 μg/mL Avns); Avns medium-dose (2 μg/mL LPS + 20 μg/mL Avns); and Avns high-dose (2 μg/mL LPS + 40 μg/mL Avns). Avns (purity ≥ 98%), obtained from Jiangxi Xuesong Natural Medicinal Oil Co., Ltd. (Ji’an, China), which contains multiple Avn monomers, were used for all cellular and enzymatic assays. Avns were dissolved in DMSO and diluted in culture medium, with a final DMSO concentration of 0.1% (*v*/*v*); the vehicle control received an equal volume of DMSO (0.1%, *v*/*v*). LPS was purchased from Sigma-Aldrich (St. Louis, MO, USA), and was dissolved in PBS and diluted in culture medium. Cells were incubated for the indicated time periods (2 h for the detection of ERK1/2, p-ERK1/2, p-c-Fos, c-Fos, IL-1β and IL-6; 24 h for COX-2) before sample collection.

#### 2.4.4. COX-2 Enzyme Activity Inhibition Assay

The inhibitory effect of Avns on COX-2 enzymatic activity was determined using a COX-2 inhibitor screening kit (Beyotime Biotechnology, Shanghai, China). COX-2 cofactor working solution, COX-2 working solution, COX-2 substrate working solution, and the positive inhibitor celecoxib solution were prepared according to the manufacturer’s instructions. The assay was conducted in a 96-well black plate. The following groups were included: a blank control (without COX-2 enzyme), a 100% enzyme activity control (with COX-2 enzyme but without inhibitor), a positive control (with COX-2 enzyme and 1 μmol/L celecoxib), and Avns treatment groups (with COX-2 enzyme and Avns at 20, 40, 80, 160 and 320 μg/mL, each in triplicate). COX-2 cofactor working solution and COX-2 working solution were added to each well according to the kit protocol, followed by the test samples (Avns or celecoxib). After mixing, the plate was incubated at 37 °C for 10 min. Then, 5 µL of COX-2 Probe was added to each well, followed immediately by 5 µL of COX-2 Substrate working solution to initiate the reaction. The plate was mixed thoroughly and incubated at 37 °C for 5 min in the dark. Fluorescence intensity was measured using a microplate reader at excitation 560 nm and emission 590 nm. The COX-2 inhibition rate was calculated as follows:

$$Inhibition\left(\%\right)=\frac{{RFU}_{100\%\;enzymeactivity}-{RFU}_{sample}}{{RFU}_{100\%\;enzymeactivity}-{RFU}_{blank}}\times 100\%$$

#### 2.4.5. PGE_2_ Quantification (ELISA)

Cells were treated with LPS with or without Avns for 24 h, then the supernatants were collected and centrifuged at 3000 r/min for 10 min at 4 °C. PGE2 levels in cell culture supernatants were measured using a PGE2 ELISA kit (Beyotime Biotechnology, Shanghai, China). Standard dilutions were prepared following the manufacturer’s instructions. Samples or standards (50 µL) were added to the precoated microplate, followed by HRP-conjugated detection antibody, and incubated at 37 °C for 60 min. After washing for 5 times, 50 µL of substrates A and B were added and incubated at 37 °C for 15 min in the dark. The reaction was terminated with 50 µL of stop solution, and absorbance was recorded at 450 nm. PGE2 concentrations were calculated from the standard curve.

#### 2.4.6. Western Blot Analysis

After treatment as described above, the supernatants were removed and cells were washed twice with PBS. Cells were then lysed with RIPA buffer containing protease and phosphatase inhibitors on ice for 30 min. Protein concentrations were determined using a BCA assay. Equal amounts of protein (20 μg) were separated by 10% SDS-PAGE and transferred onto PVDF membranes. Membranes were blocked with 5% non-fat milk in TBST for 50 min and then incubated overnight at 4 °C with primary antibodies against COX-2, ERK1/2, phospho-ERK1/2, c-Fos, IL-6, IL-1β (all from ABclonal, Wuhan, China), and phospho-c-Fos (from Selleck, Shanghai, China); β-actin was used as the loading control. After washing with TBST, membranes were incubated with HRP-conjugated secondary antibody (Beyotime, Shanghai, China) for 90 min at room temperature. Protein bands were visualized using an ECL system and quantified using ImageJ 1.53 software. Phospho-ERK1/2 and phospho-c-Fos were normalized to their corresponding total proteins (ERK1/2 and c-Fos, respectively), whereas all other proteins were normalized to β-actin.

#### 2.4.7. Statistical Analysis

All experiments were performed in three independent biological replicates, and data are expressed as mean ± standard deviation (SD). Statistical analyses were performed using SPSS 22.0 software (IBM Corporation, Armonk, NY, USA). One-way analysis of variance (ANOVA) followed by Dunnett’s post hoc test was used for multiple group comparisons. A value of *p* < 0.05 was considered statistically significant.

## 3. Results

### 3.1. Cross-Validation and Predictive Performance of the CTI Model

In this study, a dataset comprising 2398 inflammation-related CTI pairs was constructed from the DrugBank database, with an equal number of putative negative pairs generated by random sampling from the full combinatorial space. A CTI prediction model was then developed by integrating Jaccard similarity-based network construction, RWR-based topological feature extraction, DAE-based feature dimensionality reduction, and a 1D-CNN classifier. To evaluate the reliability of the proposed model, stratified 10-fold cross-validation was performed on the dataset. The model exhibited robust and consistent predictive performance across all folds, achieving a mean AUC of 0.9587 and a mean AUPR of 0.9590 (Table 1), with standard deviations of 0.0026 and 0.0025, respectively. These results indicate that the model has the potential for predicting previously unrecorded compound–target interactions.

### 3.2. Preliminary Validation of Model Applicability to Food Functional Factors

To preliminarily validate the model’s applicability to food-derived compounds, eight food functional factors representing distinct chemical classes were selected, including flavonoids (quercetin), catechins (EGCG), terpenoids (artemisinin and limonene), phenolic acids (ferulic acid), polyunsaturated fatty acids (EPA), carotenoids (β-carotene), and alkaloids (caffeine). Their SMILES structures were input into the established CTI- prediction model, and the top five predicted targets for each compound were ranked by binding probability (Table 2). The predicted targets included known and experimentally confirmed targets (e.g., PTGS2 for quercetin [40] and NFKB1 for EGCG [41]), targets with reported regulatory effects but without confirmed binding (e.g., targets of ferulic acid), as well as previously unreported targets with no documented regulatory effects, which may represent novel candidates for further investigation.

To further validate the reliability of the predictions, molecular docking was performed for the 40 compound-target pairs (8 compounds × top 5 targets). The docking results (Table 2) showed that 32 out of 40 pairs (80%) exhibited binding energies below −7.0 kcal/mol, indicating favorable predicted binding, while the remaining 8 pairs (20%) showed binding energies ranging from −4.8 to −7.0 kcal/mol, suggesting moderate binding potential. Notably, favorable binding energies were observed for quercetin with PTGS2 (−9.1 kcal/mol) and PTGS1 (−8.8 kcal/mol), artemisinin with CYP1A2 (−9.1 kcal/mol) and CYP3A4 (−9.0 kcal/mol), and β-carotene with RXRA (−8.8 kcal/mol). These results indicate that the majority of the predicted interactions exhibited favorable binding affinities, supporting the binding potential of the model-predicted targets in food functional factor target identification.

### 3.3. Avns Target Prediction and Network Analysis

#### 3.3.1. Target Prediction and Overlap Analysis

**Figure 2:**
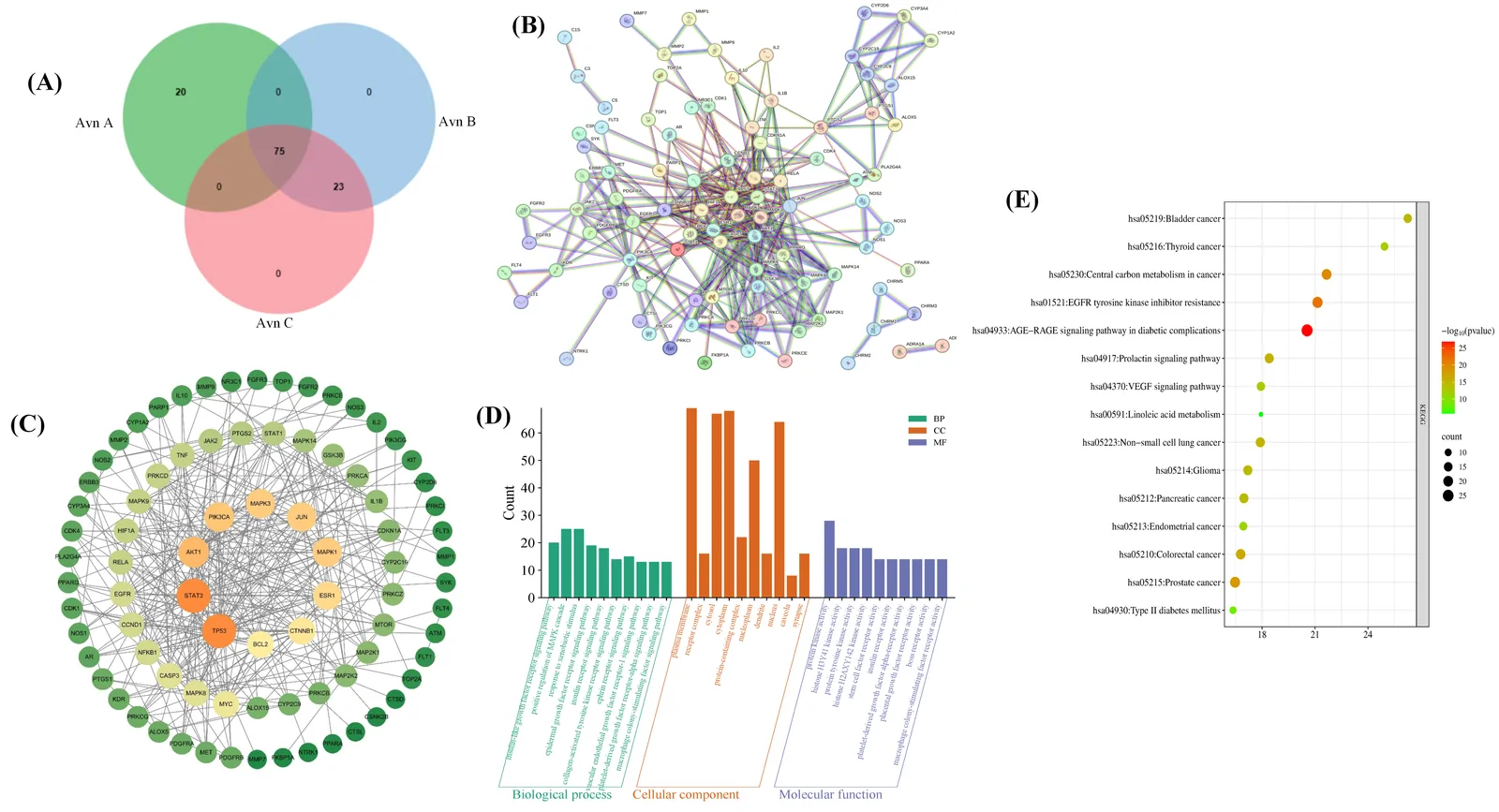
Network Analysis of predicted potential anti-inflammatory targets of Avns. (**A**) Venn diagram showing the overlap of predicted targets among Avn A, Avn B, and Avn C. (**B**) Protein–protein interaction (PPI) network of the 118 union targets. (**C**) Topological analysis and evaluation of the PPI network using Cytoscape. (**D**) Gene Ontology (GO) enrichment analysis of the core targets. The top enriched terms in Biological Process (BP), Cellular Component (CC), and Molecular Function (MF) are shown. (**E**) KEGG pathway enrichment bubble plot of the core targets. Bubble size represents gene count, and color intensity reflects −log10 (Q-value).

To further explore the potential anti-inflammatory targets of Avns, the trained CTI prediction model was applied to predict targets for the three most abundant Avn monomers: Avn A, Avn B, and Avn C. The SMILES strings of each monomer were input into the model, and the top 100 predicted targets (ranked by interaction probability) were retained for subsequent analysis. Among the top five predicted targets for all three monomers, PTGS1 (COX-1), PTGS2 (COX-2), MAPK3 (ERK1), MAPK1 (ERK2), and TNF (TNF-α) were consistently ranked with high confidence, indicating robust and convergent predictions across the three structurally related compounds. A Venn diagram was generated to visualize the overlap among the predicted targets of the three monomers (Figure 2A), yielding a total of 118 non-redundant targets across the three monomers. Among these, 75 targets (63.6%) were common to Avn A, Avn B, and Avn C, suggesting substantial target overlap. This high degree of convergence is attributable to the structurally conserved core skeleton of Avns (condensation of anthranilic acid derivatives with hydroxycinnamic acid derivatives), implying that the three monomers may exert anti-inflammatory effects through highly synergistic and overlapping mechanisms.

#### 3.3.2. PPI Network Analysis and Identification of Core Targets

The 118 non-redundant targets were imported into the STRING database to construct a protein–protein interaction (PPI) network with Homo sapiens as the background organism. Disconnected nodes were excluded, and the network was visualized and analyzed using Cytoscape 3.8.0. Topological analysis revealed that nodes with high connectivity, including MAPK1, MAPK3, STAT3, TP53, and AKT1, were predominantly localized in the central region of the network. To prioritize biologically relevant targets, genes with degree, betweenness centrality, and closeness centrality exceeding the respective median values were retained as core targets. The median-based threshold was chosen to retain nodes with above-average topological importance while maintaining a manageable number of targets for downstream analysis. Nine core targets were obtained, including STAT3, TP53, AKT1, PIK3CA, MAPK3, JUN, MAPK1, CTNNB1, and BCL2 (Figure 2B,C). These core targets are involved in several key signaling pathways, such as the MAPK pathway, PI3K-AKT pathway, and AGE-RAGE pathway.

#### 3.3.3. GO Enrichment Analysis

GO enrichment analysis was performed using Metascape to functionally annotate the nine core targets across three categories: BP, CC, and MF, with a significance threshold of *p* < 0.05 (Figure 2D). CC analysis revealed that the core targets were predominantly localized in the cytosol and organelle lumen, consistent with the intracellular distribution of signaling kinases (MAPK3/1 and AKT1) and transcription regulators (STAT3, TP53, and JUN). Enrichment of adherens and adhesion junctions was specifically associated with CTNNB1, reflecting its role in cell–cell adhesion. MF analysis showed that the core targets were primarily enriched in protein binding and RNA binding, consistent with their roles as transcription factors (STAT3, TP53, JUN) and kinases (MAPK3/1, AKT1, PIK3CA). Additional enrichment was observed in protein phosphatase binding and ubiquitin-protein ligase binding, the latter involving TP53 and BCL2, both of which are known to be regulated by ubiquitination. BP analysis indicated that the core targets were predominantly involved in phosphate metabolic processes (including phosphorylation), mediated by AKT1, PIK3CA, and MAPK3/1; cellular responses to stress and external stimuli, associated with TP53 and BCL2; and positive regulation of protein metabolic processes, reflecting the regulatory roles of these targets in inflammatory signaling cascades.

#### 3.3.4. KEGG Pathway Enrichment Analysis

KEGG pathway enrichment analysis identified 173 significantly enriched pathways, with the top 20 presented in Figure 2E. The most significantly enriched pathways were predominantly cancer-related, including bladder cancer, thyroid cancer, and non-small cell lung cancer, as well as the AGE-RAGE and EGFR tyrosine kinase inhibitor resistance pathways. These cancer-related pathways share substantial molecular overlap with inflammatory signaling, particularly through MAPK, NF-κB, and PI3K-AKT, consistent with the established role of chronic inflammation in tumorigenesis. Since the KEGG results were dominated by highly aggregated cancer-related terms, the subsequent experimental validation focused on the inflammation-related pathways identified in the GO enrichment analysis and supported by the target prediction results, particularly MAPK/ERK signaling.

### 3.4. Molecular Docking Validation of Avns Binding to COX-2 and ERK1/2

To verify the physical binding potential between Avns and the predicted core targets, molecular docking was performed. MAPK3 (ERK1) and MAPK1 (ERK2) were selected as primary docking receptors, as they were not only identified as core targets in the PPI network but also consistently ranked among the top five predicted targets for all three Avn monomers. COX-2 (PTGS2) was not among the nine core targets identified by the PPI network analysis. It was nevertheless included in the docking and experimental validation because it ranked second in our inflammation target prediction model (after COX-1). This choice was further supported by the well-established role of COX-2 in inflammatory signaling and its close functional association with the ERK pathway [42]. Avn A, Avn B, and Avn C were docked into the active sites of COX-2, MAPK3 (ERK1), and MAPK1 (ERK2). All three monomers exhibited favorable binding affinities (Table 3).

#### 3.4.1. Binding Affinity Analysis of Avns to COX-2

**Figure 3:**
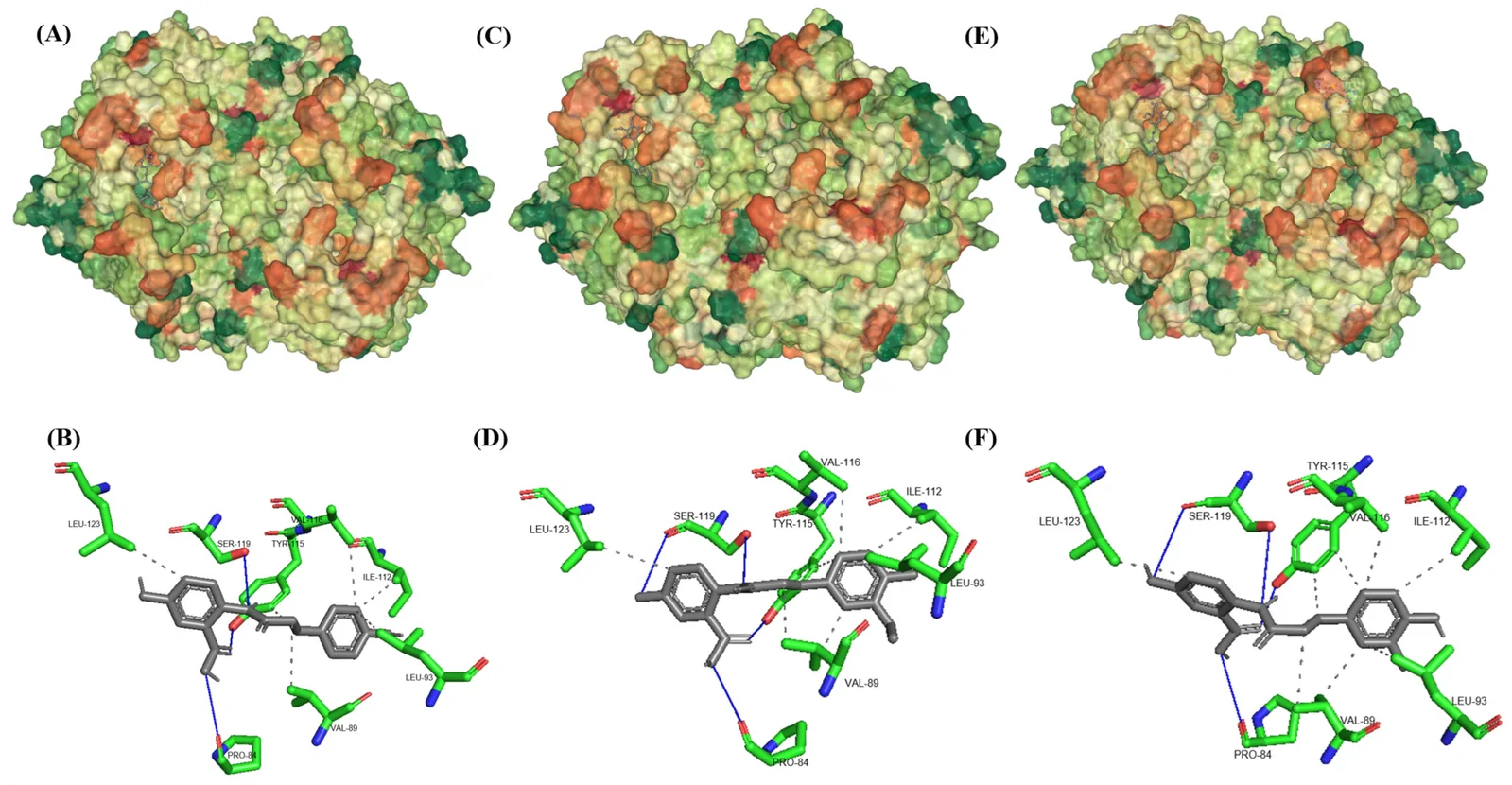
Molecular docking of Avns with COX-2. (**A**,**B**) Overview (**A**) and detailed view (**B**) of Avn A (grey) bound at the cyclooxygenase channel entrance of COX-2. (**C**,**D**) Overview (**C**) and close-up view (**D**) of Avn B (grey) in the same binding site. (**E**,**F**) Overview (**E**) and close-up view (**F**) of Avn C (grey) in the cyclooxygenase channel. Key interacting residues Pro84, Val89, Leu93, Ile112, Tyr115, Val116, Ser119, and Leu123 are shown as green sticks. Hydrogen bonds are shown as blue solid lines, and hydrophobic interactions are shown as grey dashed lines.

To evaluate the binding potential of Avns to COX-2, molecular docking was used. Avn A, Avn B, and Avn C exhibited binding energies of −7.7, −7.7, and −8.0 kcal/mol, respectively (Figure 3). All three compounds occupied the cyclooxygenase channel entrance of COX-2 (chain B), sharing an identical set of interacting residues: Pro84, Val89, Leu93, Ile112, Tyr115, Val116, Ser119, and Leu123. Hydrophobic contacts were formed with Val89, Leu93, Ile112, Tyr115, Val116, and Leu123, while hydrogen bonds were formed with Pro84, Tyr115, and Ser119. Avn C formed a hydrogen bond with Tyr115 at 2.06 Å, Avn A at 2.08 Å, and Avn B at 2.18 Å. Avn C and Avn B each formed two hydrogen bonds with Ser119, whereas Avn A formed one. Overall, all three Avn monomers exhibited favorable binding affinities to COX-2, but whether this binding translated into functional inhibition of COX-2 remains to be experimentally verified.

#### 3.4.2. Binding Affinity Analysis of Avns to MAPK3 (ERK1)

**Figure 4:**
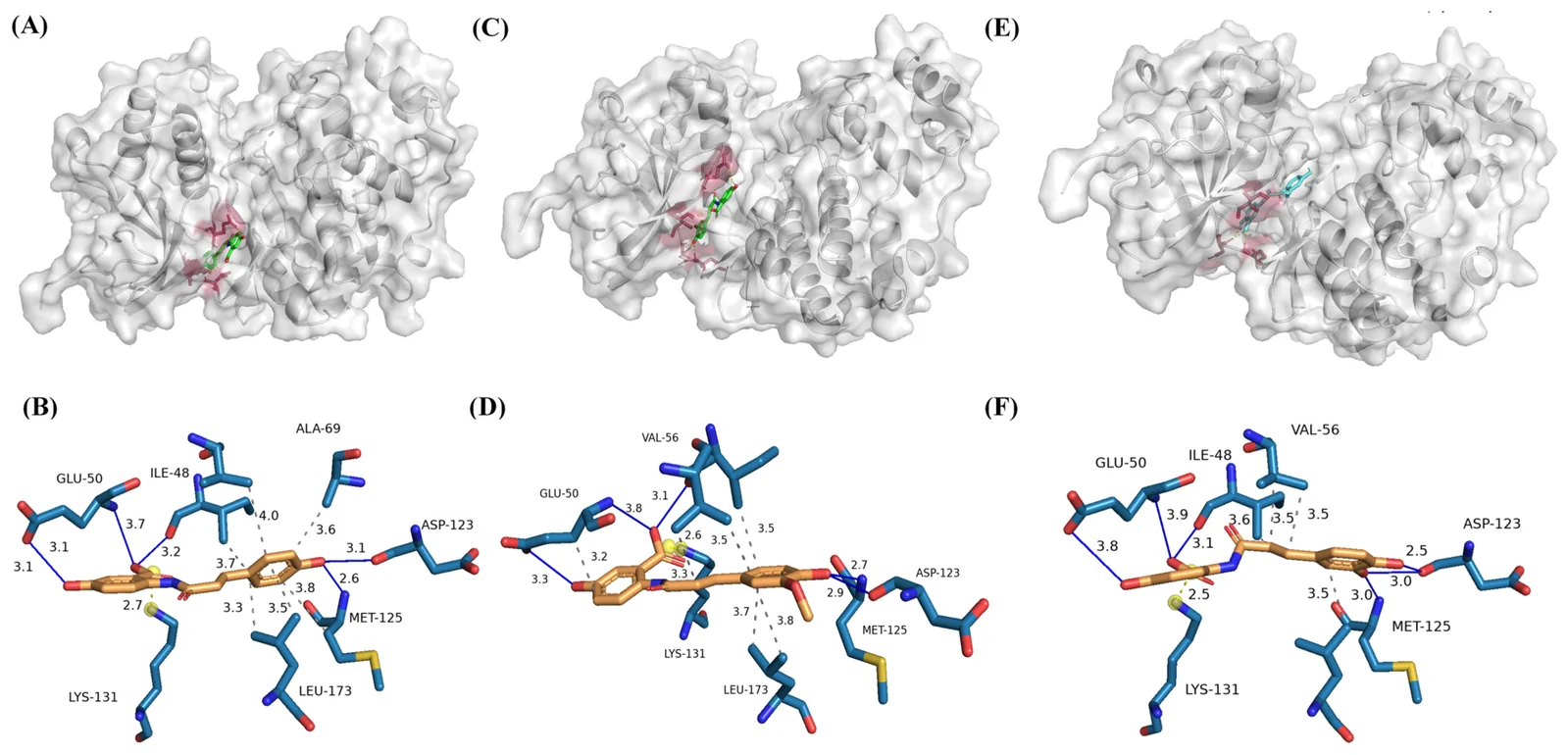
Molecular docking of Avns with MAPK3. (**A**,**B**) Overview (**A**) and detailed view (**B**) of Avn A bound at the ATP-binding pocket of MAPK3. (**C**,**D**) Overview (**C**) and close-up view (**D**) of Avn B in the same binding site. (**E**,**F**) Overview (**E**) and close-up view (**F**) of Avn C in the ATP-binding pocket. Key interacting residues Ile48, Glu50, Val56, Ala69, Asp123, Met125, Lys131, and Leu173 are shown as blue sticks (**B**,**D**,**F**). Hydrogen bonds are shown as blue solid lines, hydrophobic interactions are shown as grey dashed lines, and the salt bridge is shown as a yellow dashed line.

To examine the binding potential of Avns to MAPK3, molecular docking was performed at the ATP-binding site defined by the co-crystallized ligand 5ID. Avn A, Avn B, and Avn C exhibited binding energies of −7.1, −7.3, and −7.7 kcal/mol to MAPK3, respectively (Table 3). All three monomers occupied the ATP-binding pocket and interacted with residues in the N-lobe, the hinge region, and the C-lobe. The same key residues were engaged by all three monomers: ILE48, GLU50, VAL56, ASP123, MET125, LYS131, and LEU173. Hydrogen bonds were formed with ILE48, GLU50, ASP123, and MET125, a salt bridge was formed with LYS131, and hydrophobic contacts involved ILE48, GLU50, VAL56, and LEU173. Avn A additionally formed a hydrophobic contact with ALA69. All three monomers adopted energetically favorable binding modes within the ATP-binding pocket (Figure 4). Whether these predicted binding modes have biological significance requires experimental validation.

#### 3.4.3. Binding Affinity Analysis of Avns to MAPK1 (ERK2)

**Figure 5:**
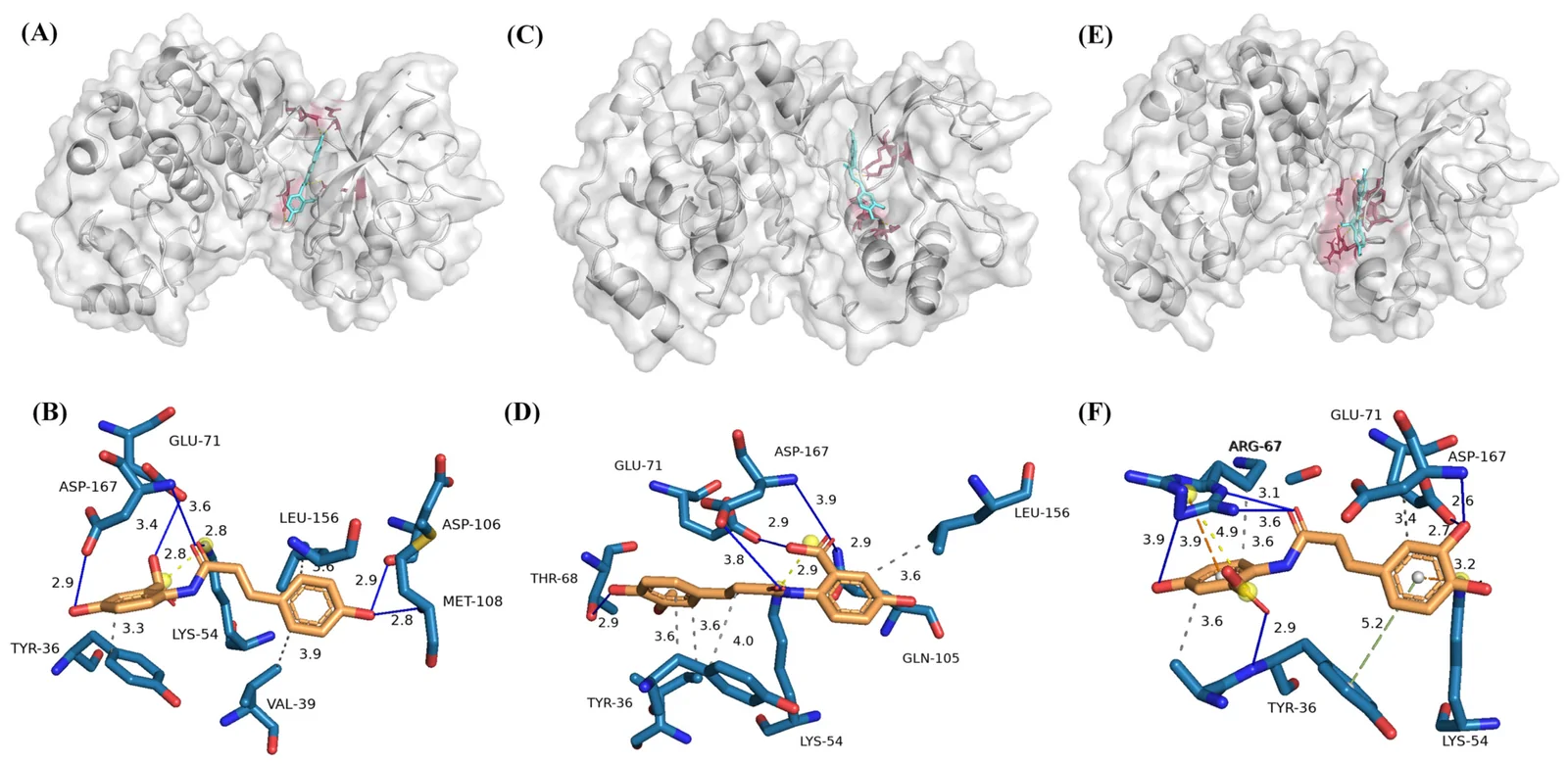
Molecular docking of Avns with MAPK1 (ERK2). (**A**,**B**) Overview (**A**) and detailed view (**B**) of Avn A bound at the ATP-binding pocket of MAPK1, with key interactions involving Lys54, Glu71, Asp106, Met108, and Asp167. (**C**,**D**) Overview (**C**) and close-up view (**D**) of Avn B bound to the same binding site, with contacts involving Tyr36, Lys54, Thr68, Glu71, Gln105, and Asp167. (**E**,**F**) Overview (**E**) and close-up view (**F**) of Avn C bound at the ATP-binding pocket, with interactions at Tyr36, Lys54, Arg67, Glu71, and Asp167. Key interacting residues are shown as blue sticks. Hydrogen bonds are shown as blue solid lines, hydrophobic interactions as grey dashed lines, and the salt bridge as a yellow dashed line. π-stacking and π-cation interactions are shown as green and orange dashed lines, respectively.

To evaluate the binding potential of Avns to MAPK1, molecular docking was employed at the ATP-binding site defined by the co-crystallized ligand. Avn A, Avn B, and Avn C exhibited binding energies of −7.3, −7.0, and −7.5 kcal/mol to MAPK1, respectively (Table 3). All three monomers occupied the ATP-binding pocket and interacted with residues in the N-lobe, the hinge region, and the C-lobe. The same key residues were engaged by all three monomers: TYR36, LYS54, GLU71, and ASP167. Hydrogen bonds were formed with LYS54, GLU71, and ASP167, a salt bridge was formed with LYS54, and hydrophobic contacts involved TYR36, ILE56, and LEU156. Avn A additionally interacted with ASP106 and MET108 in the hinge region, whereas Avn B additionally interacted with THR68 and GLN105, and Avn C additionally interacted with ALA35 and ARG67. For Avn C, π-stacking with TYR36 and π-cation interactions with LYS54 and ARG67 were also observed (Figure 5). All three monomers adopted energetically favorable binding modes within the ATP-binding pocket. The apparent differences in contact residues between MAPK3 and MAPK1 largely reflect the different numbering systems of the two kinases; structurally equivalent positions (e.g., ASP123/MET125 in MAPK3 corresponding to ASP106/MET108 in MAPK1) are engaged in both cases. Further experimental work is required to determine whether these predicted interactions are functionally relevant.

### 3.5. The Effect of Avns on Inhibiting COX-2 Activity and Expression

**Figure 6:**
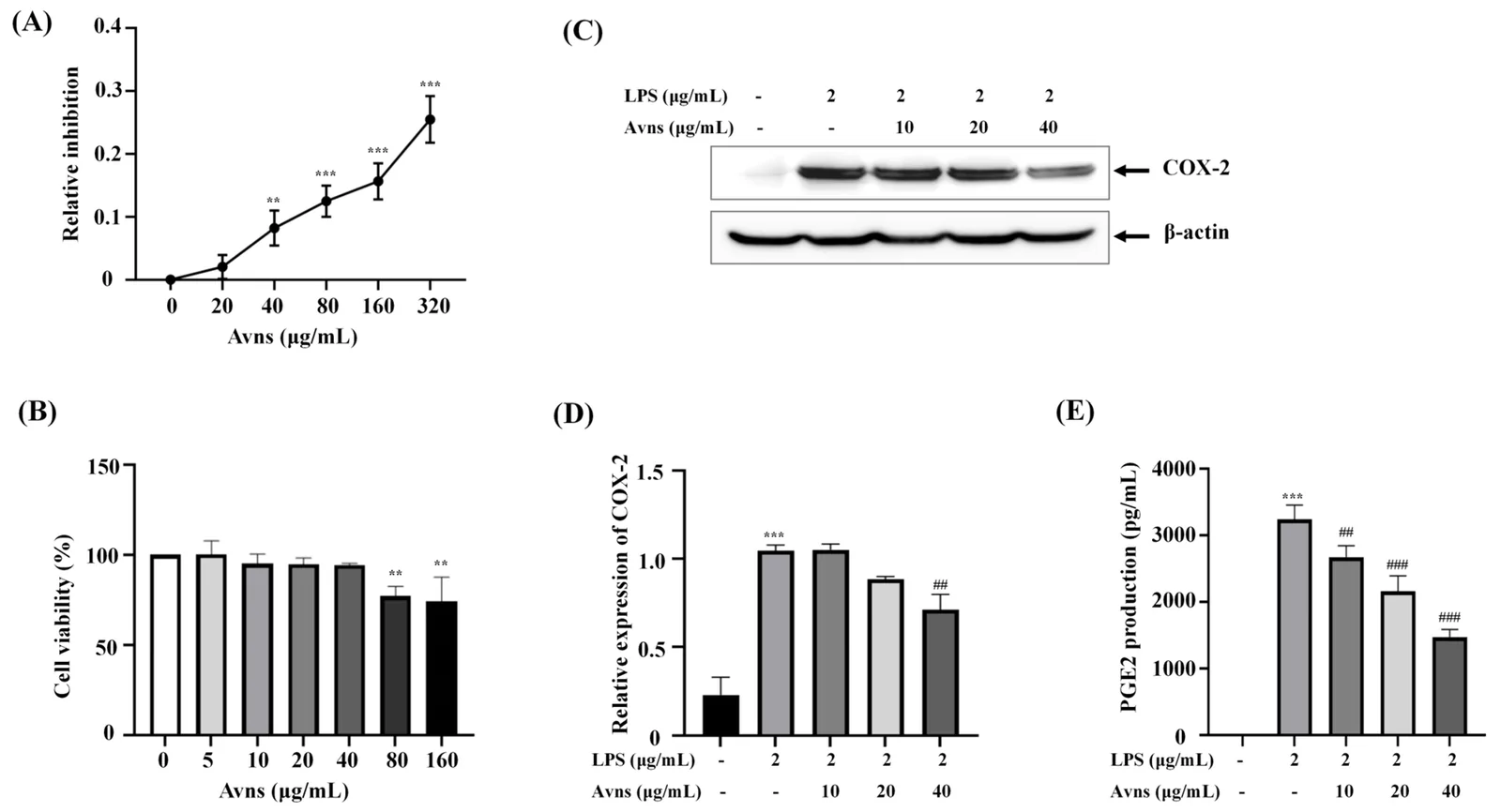
Effects of Avns on COX-2 activity and expression in RAW264.7 macrophages. (**A**) COX-2 enzyme activity inhibition assay. Avns inhibited COX-2 activity in a dose-dependent manner in a cell-free system. ** *p* < 0.01 and *** *p* < 0.001 vs. 100% enzyme activity control. (**B**) Cell viability assay (CCK-8). RAW264.7 cells were treated with Avns (0, 5, 10, 20, 40, 80, and 160 μg/mL) for 24 h. ** *p* < 0.01 vs. control. (**C**,**D**) Representative bands (**C**) and quantification (**D**) of COX-2 protein expression. Western blot analysis was performed after cells were stimulated with LPS (2 μg/mL) and treated with Avns (0, 10, 20, and 40 μg/mL) for 24 h. *** *p* < 0.001 vs. control group (LPS-, Avns-); ^##^ *p* < 0.01 vs. LPS treated alone. (**E**) PGE_2_ production in cell culture supernatants was measured by ELISA after cells were stimulated with LPS (2 μg/mL) and treated with Avns (0, 10, 20, and 40 μg/mL) for 24 h. *** *p* < 0.001 vs. control group (LPS-, Avns-); ^##^ *p* < 0.01 and ^###^ *p* < 0.001 vs. LPS treated alone. Data are presented as mean ± SD (*n* = 3 independent biological replicates).

To determine whether the predicted binding of Avns to COX-2 translates into functional inhibition, we measured COX-2 enzymatic activity in the presence of Avns at varying concentrations using a COX-2 inhibitor screening kit. The Avns inhibited COX-2 activity in a dose-dependent manner, reaching approximately 25% at 320 µg/mL (Figure 6A). Under the same assay conditions, celecoxib at 1 μM inhibited COX-2 activity by 49.01 ± 1.77%.

We next performed cellular experiments to further evaluate the effects of Avns on COX-2 expression and activity. The cytotoxic profile of Avns was first assessed in RAW264.7 macrophages using the CCK-8 assay. As presented in Figure 6B, no significant cytotoxicity was observed at concentrations ranging from 5 to 40 μg/mL compared to the control group. However, cell viability decreased markedly at 80 and 160 μg/mL (*p* < 0.01). Accordingly, 10, 20, and 40 μg/mL were selected as non-cytotoxic intervention doses for subsequent experiments.

Having established the safe dose range of Avns in macrophages, we next examined whether Avns affected COX-2 protein expression. RAW264.7 macrophages were stimulated with LPS 2 μg/mL in the presence or absence of Avns, and COX-2 expression was analyzed by Western blotting assay (Figure 6C,D). In the absence of LPS stimulation, COX-2 expression was minimal in the control group while LPS stimulation markedly upregulated COX-2 protein levels compared to the control group (*p* < 0.001). Avns intervention suppressed COX-2 induction in a dose dependent manner. Specifically, 40 μg/mL Avns significantly reversed this upregulation (*p* < 0.01), suggesting that Avns dramatically affected COX-2 expression.

PGE2, a key downstream inflammatory mediator, is synthesized from PGH2 by PGE synthases. Although COX-1 also contributes PGH2, COX-2 is the inducible and predominant source of PGE2 under inflammatory conditions. Given the dose-dependent suppression of COX-2 protein expression by Avns, we next examined whether this reduction was accompanied by decreased production of PGE2. As shown in Figure 6E, LPS stimulation led to a marked increase in PGE2 production (*p* < 0.001), whereas Avns treatment resulted in a dose-dependent suppression of PGE_2_ release. Collectively, these findings demonstrate that Avns suppressed COX-2 at both the activity and protein expression levels, thereby attenuating LPS-induced inflammatory responses in macrophages.

### 3.6. Avns Suppress the ERK/c-Fos Inflammatory Signaling Axis

**Figure 7:**
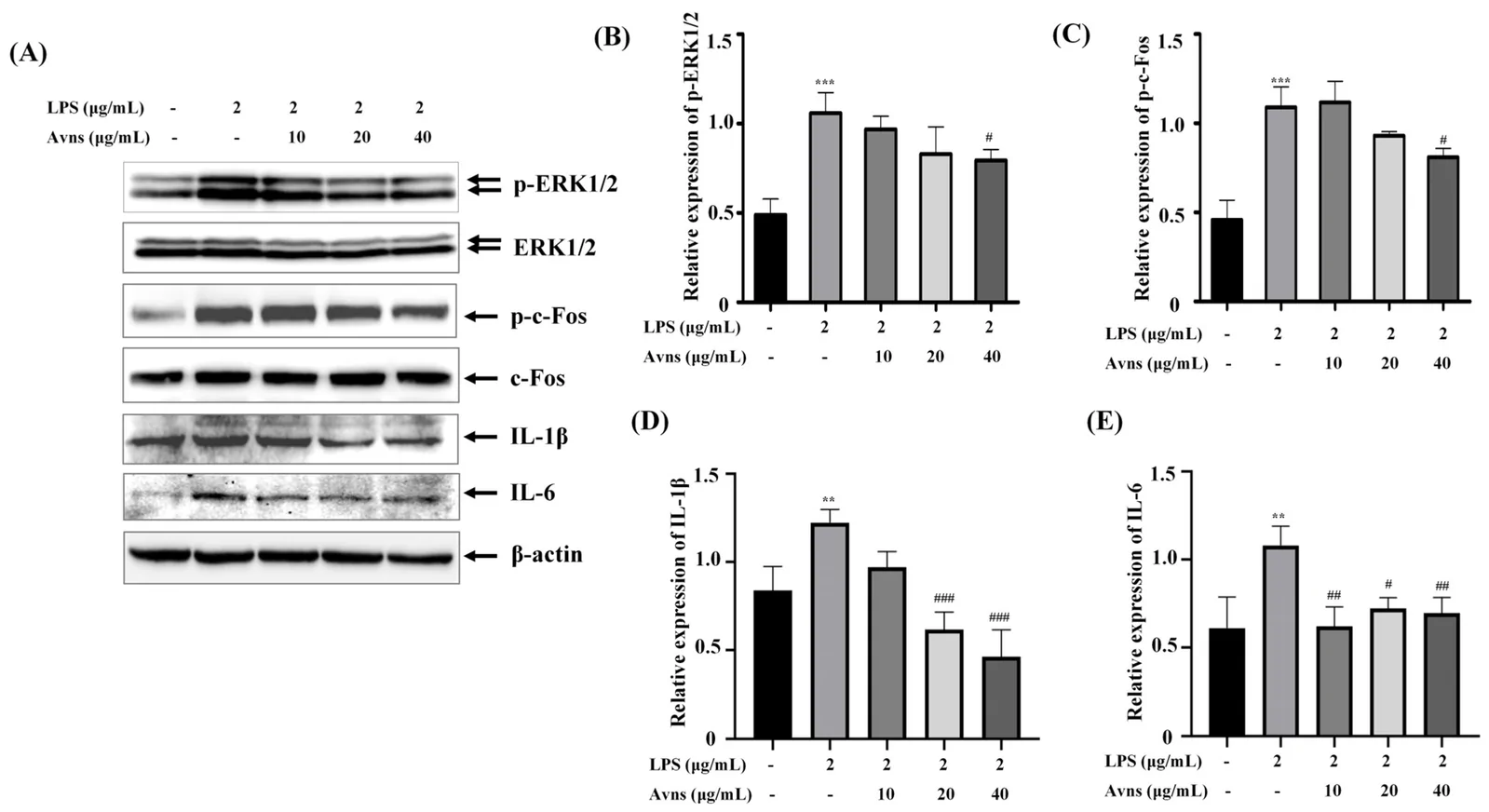
Avns suppress the ERK/c-Fos inflammatory signaling axis in LPS-stimulated RAW264.7 macrophages. (**A**) Representative Western blot bands of p-ERK1/2, ERK1/2, p-c-Fos, c-Fos, IL-1β, IL-6, and β-actin in LPS-stimulated RAW264.7 macrophages treated with Avns (0, 10, 20, and 40 μg/mL) for 2 h. (**B**–**E**) Quantification of p-ERK1/2 (**B**), p-c-Fos (**C**), IL-1β (**D**), and IL-6 (**E**) protein levels. Data are shown as mean ± SD (*n* = 3 independent biological replicates). ** *p* < 0.01, and *** *p* < 0.001 vs. Control group (LPS-, Avns-); ^#^ *p* < 0.05, ^##^ *p* < 0.01, and ^###^ *p* < 0.001 vs. LPS treated alone.

As kinases, the activity of ERK1/2 is primarily reflected by their phosphorylation levels. Having established the strong binding potential between Avns and ERK1/2 through molecular docking, we next examined whether Avns functionally affect ERK activity, and Western blotting was performed to detect the phosphorylation levels of ERK1/2 (p-ERK1/2) and total ERK1/2 in RAW264.7 macrophages treated with different concentrations of Avns (Figure 7A,B). In the absence of LPS stimulation, p-ERK1/2 remained at a low basal level. Upon stimulation with 2 μg/mL LPS, p-ERK1/2 expression was dramatically upregulated (*p* < 0.001), indicating strong activation of the MAPK pathway. Treatment with Avns suppressed LPS-induced ERK1/2 hyperphosphorylation in a dose-dependent manner, with significant inhibition observed at 40 μg/mL (*p* < 0.05).

As a downstream transcription factor of the ERK signaling pathway, c-Fos is activated through phosphorylation by activated ERK [43]. To determine whether the inhibition of ERK phosphorylation by Avns led to suppression of downstream transcriptional activation, we next examined the phosphorylation levels of c-Fos (p-c-Fos) by Western blotting (Figure 7A,C). In the absence of LPS stimulation, p-c-Fos expression was barely detectable. LPS challenge markedly upregulated p-c-Fos expression compared to the control group (*p* < 0.001), whereas Avns treatment significantly suppressed this upregulation at 40 μg/mL (*p* < 0.05). This suppression was highly consistent with the inhibition of upstream ERK1/2 phosphorylation, indicating that Avns effectively blocked the signal transduction from the cytoplasm to the nucleus, thereby attenuating the transcriptional activation of pro-inflammatory genes.

To further evaluate whether the inhibition of the ERK/c-Fos signaling pathway by Avns led to reduced expression of downstream pro-inflammatory cytokines, we next examined the protein levels of mature IL-1β (IL-1β) and IL-6 by Western blotting (Figure 7A,D,E). In the absence of LPS stimulation, IL-1β and IL-6 expression was minimal. LPS markedly upregulated both cytokines (*p* < 0.01), whereas Avns treatment significantly suppressed their expression. These results demonstrated that Avns effectively inhibited the production of key inflammatory mediators, further confirming that the blockade of upstream ERK1/2 phosphorylation and downstream c-Fos activation resulted in phenotypic suppression of inflammation at the cellular level.

## 4. Discussion

Chronic low-grade inflammation is a common pathological basis for various chronic diseases. Identifying anti-inflammatory food functional factors from dietary sources represents a promising strategy for disease prevention, yet their molecular targets remain largely unidentified. In this study, we developed a predictive model integrating deep learning and bioinformatics for inflammation-related target identification of food functional factors. The model showed robust performance with a mean AUC of 0.9587 and AUPR of 0.9590 in cross-validation, and was applied to predict targets of eight representative food compounds, with molecular docking suggesting favorable binding to their top five predicted targets, providing preliminary support for the model. We further used the model to predict the potential anti-inflammatory targets of Avns. The top five predicted targets with the highest binding probabilities were PTGS2 (COX-2), MAPK3 (ERK1), MAPK1 (ERK2), STAT3, and AKT1. Three major monomers of Avns (Avn A, B, C) yielded 118 potential targets in total. PPI network analysis identified nine hub targets, including STAT3, TP53, AKT1, PIK3CA, MAPK3, JUN, MAPK1, CTNNB1, and BCL2, and GO/KEGG enrichment analyses revealed involvement in MAPK, PI3K-AKT, and AGE-RAGE signaling pathways. Molecular docking suggested favorable binding of Avn monomers to COX-2 and ERK1/2. In vitro experiments showed that Avns dose-dependently inhibited COX-2 enzymatic activity, suppressed COX-2 expression and PGE_2_ release, and reduced ERK1/2 phosphorylation, c-Fos activation, and IL-6/IL-1β expression in LPS-stimulated RAW264.7 macrophages. Collectively, these findings suggest that Avns may exert anti-inflammatory effects through a multi-target mechanism involving COX-2 inhibition and blockade of the ERK/c-Fos signaling axis, providing new evidence for the molecular targets of Avns and offering a potentially useful strategy for target screening of other food functional factors.

Most dietary bioactive compounds resemble pharmaceutical agents in both chemical structure and biological activity [44]. Drug molecules and food-derived small molecules are both organic compounds of low molecular weight, and their interactions with protein targets are determined by shared physicochemical determinants, including molecular weight, hydrogen-bonding capacity, and aromatic ring features [45]. Training a deep learning model on drug-target interaction data does not simply learn a catalog of drug-specific associations. Instead, the model captures the relationship between small-molecule structural features and target binding, a relationship that generalizes beyond known drugs and extends to other small molecules, including those from food sources [46]. CNN has been widely demonstrated to be effective in extracting local patterns from sequential data [47]. RWR has been shown to be capable of capturing long-range dependencies in biological networks [48], and DAE has been proven effective in learning robust feature representations from noisy data [49]. DrugBank provides a large collection of experimentally verified compound-target interactions with curated bioactivity data, offering a reliable data foundation for model training [23]. Accordingly, RWR, DAE, and 1D-CNN were adopted as a combined strategy to address the sparsity and noise inherent in food-derived compound-target interaction data. The 10-fold cross-validation results (AUC = 0.9587, AUPR = 0.9590), together with molecular docking and cellular assay validations, confirmed the reliability and predictive accuracy of the model.

The predicted targets were consistent with previously reported compound-target interactions. Among the 40 predicted compound-target pairs, known associations were successfully recapitulated. For instance, quercetin exhibited strong binding affinity to PTGS2 (COX-2, −9.1 kcal/mol) and PTGS1 (COX-1, −8.8 kcal/mol), consistent with its well-documented role as a dual COX inhibitor [50]. EGCG showed favorable binding to NFKB1 (−7.7 kcal/mol), aligning with its known regulatory effect on the NF-κB signaling pathway [51]. The successful identification of these established interactions confirms the model’s ability to recover biologically meaningful relationships from compound structural features. Beyond these known associations, the predictions also identified previously unreported targets with strong binding affinities. Artemisinin showed high-affinity binding to CYP1A2 (−9.1 kcal/mol) and CYP3A4 (−9.0 kcal/mol), suggesting potential interactions with cytochrome P450 enzymes that may influence its pharmacokinetic profile. β-Carotene exhibited strong binding to RXRA (−8.8 kcal/mol), a nuclear receptor involved in retinoid signaling and immune regulation, implying a possible direct regulatory role beyond its classical function as a vitamin A precursor. Limonene showed moderate binding to TRPV3 (−7.1 kcal/mol), a thermo-sensitive ion channel implicated in inflammatory pain, representing a potential novel mechanism for its reported anti-inflammatory effects. These newly identified targets, particularly those with strong binding energies, need further experimental validation and may provide new directions for understanding the molecular mechanisms of food functional factors and guiding the development of targeted nutritional interventions.

Previous work has linked Avns to the COX-2 and ERK1/2 pathways, but largely on the basis of downstream activity changes [52,53]. Whether these effects reflect binding to the target proteins or are secondary to changes elsewhere in the signaling network has remained unclear. Here we combined structure-based computational screening with biochemical validation to address this question. Our results suggest that Avns may bind at the cyclooxygenase channel entrance site of COX-2 and the ATP-binding groove of ERK1/2, thereby interfering with the phosphorylation cascade required for c-Fos activation. The predicted occupancy of both targets may not only contribute to the observed concentration-dependent inhibition of PGE_2_ synthesis, but also sever the link between COX-2-generated lipid signals and ERK-dependent transcriptional amplification, thereby interrupting a positive feedback loop implicated in the maintenance of chronic inflammatory states.

It should be noted, however, that the Avn mixture only modestly inhibited COX-2 activity in the cell-free assay, reaching approximately 25% at 320 µg/mL without attaining 50%, so that an IC_50_ could not be determined. This suggests that the cellular anti-inflammatory effects of Avns may involve additional targets beyond COX-2. Consistent with this, these effects are likely to arise from coordinated modulation of multiple targets, including the MEK–ERK–c-Fos axis, as supported by our computational predictions and cellular experiments. The discrepancy between the effective concentrations in the enzymatic and cellular assays further reflects the different nature of the two systems, and the possibility that Avns act through additional, as yet unidentified targets cannot be excluded.

While existing target deconvolution efforts for Avns have centered on cancer-related proteins, whether these dietary compounds use a similar binding strategy in inflammation has remained unexplored. Our work addresses this question by testing COX-2 and ERK1/2 as binding candidates in the inflammatory response. Interestingly, the tumor-promoting roles of COX-2 and ERK1/2 are well established, and chronic inflammation is a recognized driver of carcinogenesis [54]. Thus, the newly identified anti-inflammatory targets may also partially explain the chemopreventive properties of Avns. Furthermore, our PPI network and KEGG enrichment analyses implicated other core targets such as STAT3, AKT1, and PIK3CA, suggesting broader polypharmacological effects that warrant further experimental validation.

Computationally, the model was evaluated by random 10-fold cross-validation, which may overestimate performance when the same compound or target appears in both training and test folds. Compound-wise or target-wise splitting would provide a more stringent assessment, and the reported AUC/AUPR values may therefore be optimistic. Moreover, no published baseline was benchmarked under an identical protocol. Molecular docking against predicted targets provided complementary, though not equivalent, support for the predictions (Section 3.2, Table 2). Experimentally, Avns, like other catechol- or phenol-containing polyphenols, may undergo oxidative degradation under cell culture conditions, and such degradation products could potentially contribute to apparent COX-2 inhibition. In addition, because the COX-2 assay kit reports activity through a peroxidase-coupled readout, phenolic compounds may act as reducing cosubstrates in this system, and the modest inhibition observed may therefore partly reflect assay interference rather than true enzyme blockade. Finally, docking used human COX-2 and ERK1/2 structures while validation was performed in murine RAW264.7 cells; although both targets are highly conserved, species-specific differences in binding affinity cannot be excluded. The interpretation of ERK1/2 should also remain cautious: reduced phosphorylation supports modulation of ERK signaling, but does not by itself demonstrate direct target engagement.

## 5. Conclusions

In this study, we have developed a deep learning-based approach to predict inflammation-related targets of food functional factors, and our results suggest that COX-2 and ERK1/2 are computationally prioritized candidate targets of Avns, supported by concordant cellular responses. Although this study has several limitations, the present work provides a molecular basis for understanding the anti-inflammatory effects of Avns and illustrates the potential of artificial intelligence in the discovery and mechanistic characterization of bioactive food components.

## Figures and Tables

**Table 1:** Results of 10-Fold Cross-Validation.

Fold	AUC	AUPR
1	0.9612	0.9625
2	0.9556	0.9548
3	0.9601	0.9613
4	0.9578	0.9572
5	0.9564	0.9581
6	0.9623	0.9619
7	0.9547	0.9554
8	0.9589	0.9597
9	0.9615	0.9602
10	0.9582	0.9591
Mean ± SD	0.9587 ± 0.0026	0.9590 ± 0.0025

**Table 2:** Molecular docking of food-derived bioactive compounds with their potential anti-inflammatory targets.

Compound	Predicted Target (Gene Symbol)	Binding Affinity (kcal/mol)
Quercetin	ESR1	−8.9
Quercetin	PPARG	−8.4
Quercetin	COX-1	−8.8
Quercetin	COX-2	−9.1
Quercetin	ALOX5	−8.2
EGCG	HDAC1	−7.1
EGCG	HDAC3	−7.5
EGCG	HDAC4	−7.1
EGCG	HDAC11	−6.9
EGCG	NFKB1	−7.7
Artemisinin	ATP1A3	−7.4
Artemisinin	FTH1	−7.4
Artemisinin	CYP3A4	−9.0
Artemisinin	CYP1A2	−9.1
Artemisinin	CYP2C9	−8.1
Ferulic acid	NFKB1	−4.8
Ferulic acid	RELA	−5.3
Ferulic acid	MMP9	−6.2
Ferulic acid	ALOX5	−5.6
Ferulic acid	COX-1	−6.9
EPA	COX-1	−7.4
EPA	COX-2	−7.3
EPA	ALOX5	−7.9
EPA	PLA2G1B	−6.2
EPA	PLA2G4A	−5.9
β-Carotene	RARA	−7.5
β-Carotene	RXRA	−8.8
β-Carotene	VDR	−7.5
β-Carotene	NR1I2	−6.6
β-Carotene	PPARG	−8.2
Caffeine	ADORA2a	−5.3
Caffeine	ADORA2b	−5.2
Caffeine	ADORA1	−5.2
Caffeine	ADORA3	−6.1
Caffeine	PDE4D	−5.8
Limonene	AHR	−6.2
Limonene	TRPV3	−7.1
Limonene	S1PR5	−6.5
Limonene	TRPV2	−4.8
Limonene	KCNK3	−6.0

**Table 3:** Binding affinity of Avns to COX-2, ERK1 and ERK 2 by molecular docking analysis.

Target Protein	Avn A (kcal/mol)	Avn B (kcal/mol)	Avn C (kcal/mol)
COX-2 (PTGS2)	−7.7	−7.7	−8.0
ERK1 (MAPK3)	−7.1	−7.3	−7.7
ERK2 (MAPK1)	−7.3	−7.0	−7.5

## Data Availability

The original contributions presented in this study are included in the article. Further inquiries can be directed to the corresponding authors.

## Abbreviations

The following abbreviations are used in this manuscript:

AUC	Area Under the Curve
AUPR	Area Under the Precision-Recall Curve
Avns	Avenanthramides
BP	Biological Process
CC	Cellular Component
CNN	Convolutional Neural Network
COX-2	Cyclooxygenase-2
CTI	Compound-Target Interaction
DAE	Denoising Autoencoder
EGCG	Epigallocatechin Gallate
EPA	Eicosapentaenoic Acid
ERK	Extracellular Signal-Regulated Kinase
GO	Gene Ontology
IL-1β	Interleukin-1β
IL-6	Interleukin-6
KEGG	Kyoto Encyclopedia of Genes and Genomes
LPS	Lipopolysaccharide
MF	Molecular Function
RWR	Random Walk with Restart
